# Impact of digital economic development and environmental pollution on residents’ health: an empirical analysis based on 279 prefecture-level cities in China

**DOI:** 10.1186/s12889-023-15788-4

**Published:** 2023-05-26

**Authors:** Yan-Ting He, Yue-Chi Zhang, Wen Huang, Ruo-Nan Wang, Luo-Xuan He, Bei Li, Yi-Li Zhang

**Affiliations:** 1 School of Health Management, Southern Medical University, Guangzhou, 510515 China; 2 School of Social & Political Sciences, College of Social Sciences, University of Glasgow, Glasgow, UK; 3 The Fifth Affiliate Hospital of Southern Medical University, Guangzhou, China

**Keywords:** Digital economic development, Environmental pollution, Residents’ health, Mediating effect, Spatial Durbin model

## Abstract

**Background:**

The digital economy based on the internet and IT is developing rapidly in China, which makes a profound impact on urban environmental quality and residents’ health activities. Thus, this study introduces environmental pollution as a mediating variable based on Grossman’s health production function to explore the impact of digital economic development on the health of the population and its influence path.

**Methods:**

Based on the panel data of 279 prefecture-level cities in China from 2011 to 2017, this paper investigates the acting mechanism of digital economic development on residents’ health by employing a combination of mediating effects model and spatial Durbin model.

**Results:**

The development of digital economy makes direct improvement on residents’ health condition, which is also obtained indirectly by means of environmental pollution mitigation. Besides, from the perspective of spatial spillover effect, the development of digital economy also has a significant promoting effect on the health of adjacent urban residents, and further analysis reveals that the promoting effect in the central and western regions of China is more pronounced than that in the eastern region.

**Conclusions:**

Digital economy can have a direct promoting effect on the health of residents, and environmental pollution has an intermediary effect between digital economy and residents’ health; At the same time, there is also a regional heterogeneity among the three relationships. Therefore, this paper believes that the government should continue to formulate and implement scientific digital economy development policies at the macro and micro levels to narrow the regional digital divide, improve environmental quality and enhance the health level of residents.

## Background

“Digital Economy” is considered as economic and social activities based on and derived from new technologies such as the internet, big data, and cloud computing [1]. It is often seen as a key feature of the fourth Industrial Revolution (also known as “Industry 4.0”), and the world is in the wave of industry 4.0 reform [2]. In response to the changing situation brought about by the digital revolution, governments are taking active measures: For example, German companies are actively pushing for the establishment of external information physical systems (CPS) and internal asset information management systems (AAS) [3]; American companies responded to the fourth industrial revolution by forming an “Industrial Internet Alliance“ [4]. In China, the government has issued “Made in China 2025”, “Industrial Internet Development Action Plan (2018–2020)” and other Chinese versions of industry 4.0 programmatic policy documents to promote the development of China’s digital economy [5, 6]. The development of digital economy plays an important role in improving individual health level and improving environmental pollution. Many literatures have explored the relationship between digital economy and environmental pollution, or between digital economy and residents’ health.

Firstly, the relationship between the digital economy development and environmental pollution is studied. According to the theory of ecological modernization, digital technologies can alleviate environmental problems, which lays a theoretical foundation for examining the role of the development of the digital economy in ameliorating environmental pollution [7]. In practice, with the emergence of the energy crisis and the increasing demand for human sustainable development, the improvement effect of digital technology on the ecological environment has been the focus of society’s attention. We review the existing studies and find that many scholars have done in-depth research on the relationship between the digital economy and environmental sustainability, but their views are diverse. Firstly, most scholars agree that the digital economy can improve the quality of the environment. For example, research by Ozcan B and Ishida H found that in the development of the digital economy, related industries can reduce environmental pollution by optimizing production processes, increasing the efficiency of technological innovation, and reducing energy consumption [8, 9]. Lin B focused on the impact of the digital economy on air pollutant emissions and found that the digital economy could reduce carbon emissions by promoting industrial upgrading and technological innovation [10]. In addition, the technological effects inherent in the digital economy in traditional manufacturing can stimulate enterprises to make full use of cutting-edge technologies and promote structural dividends to improve the environment [11]; This environmental improvement effect is not only manifested in accelerating the rate of emission reduction in local, but also has a spatial spillover effect on the environmental quality of neighboring areas [12]. Secondly, with the deepening of research, some scholars have discovered that there may be " green blind spots " in the development of digital economy, for example, some studies believe that the digital economy is based on Internet technology, and the excessive use of such technologies increases the consumption of resources and energy in daily life and production, which will be detrimental to the improvement of environmental quality [13]. Although the digital economy is a new economic form, due to the peculiarities of its industry, the information technology utilized may also cause adverse consequences for waste of resources and damage to the environment [14]. What’s more, some studies have verified the existence of an “Environmental Kuznets (EKC)” curve relationship between the digital economy and environmental quality, for example, a provincial panel data survey in China found a significant “inverted U-shape” relationship between Internet development and Chinese pollution [15]. In spite of the controversial relationship between the digital economy and environmental pollution mentioned above, most scholars have not completely denied the role of digital technology in improving the environment. Generally speaking, with the deep integration and application innovation of digital technology in the fields of resources, energy and environment, the digital economy will be able to reshape the production systems, promote industrial digitalization, and directly or indirectly reduce energy consumption in the production field to improve environmental quality and promote socio-ecological sustainable development.

Secondly, the relationship between the digital economy the development and residents’ health is studied. With the construction and gradual improvement of cyber-infrastructure, medical and health service providers can provide in a systematic way targeted life cycle services to residents more rapidly through cyber-infrastructure, thus improving their health conditions. Therefore, scholars have gradually focused their studies on the impact of the application of internet and IT on residents’ health. Currently, there are two main views which are quite opposite to each other. Some scholars think that users of digital economy technologies generally have better physical and mental health and medical information decision-making abilities than non-users. What’s more, the use of the internet can promote self- evaluation of health [16], reduce depression [17], and bring about good interpersonal relationships and health behaviors [18]. In addition, another scholar proposed the concept of health 4.0 according to the concept of Industry 4.0, hoping to improve the efficiency of health service processes and medical personnel as well as the utilization rate of health services with the help of digital technology in the field of health care, and formulate targeted medical service guidelines based on big data information [19].Other scholars believe that the development of digital economy inhibits the improvement of residents’ health [20]. Among them are the supporters of techno-stress theory who hold the view that residents are unequipped for the rapid development of internet technology, resulting in some adaptive diseases [20]. For example, Azher et al. [21] found that long-term use of internet applications had a significant inhibitory effect on the physical and mental health of respondents. Internet technology provides residents with multiple forms of entertainment and information transmission channels and makes them more likely to be addicted to the internet, with their health neglected and even deteriorated, especially when the Covid-19 pandemic restricts their travel to a certain extent. Besides, excessive use of the internet can also lead to other physical and mental problems such as difficulty in falling asleep [22], anxiety [23]and aggravation of neurological diseases [24]. In summary, we can find that most scholars agree that the development of digital economy can promote the healthy development of residents, while the negative effect on health mainly lies in the degree of irrational use of digital economy technologies.

Finally, the relationship between environmental pollution and the health of the population has also been studied a lot at home and abroad. Some scholars believe that environmental pollution has been an important cause of certain chronic diseases. For example, Tsai et al. [25] found that environmental pollutants including heavy metals, air pollutants, phthalates and melamine may increase the risk of chronic kidney disease in the population. The increase of industrial sulfur dioxide emissions in some cities will lead to increased mortality of lung cancer and respiratory diseases in local and adjacent areas [26]. In addition to the negative effects of environmental pollution on respiratory diseases, it also has adverse effects on cardiovascular diseases [27] and sleep quality [28]. Besides, pollutant emission is also one of the burdens of public health expenditure in a region and country [29]. For instance, in a global long-term dynamic study on health care expenditure and environmental pollution, it was found that for every 1% increase in carbon dioxide emissions, the health expenditure of each country increases by 2.5% [30]. Economic growth in most developing countries is accompanied by an increase in energy consumption, which eventually leads to a deterioration in air quality that poses a threat to human health, and in turn, the demand for health care is increasing as a result of improved socio-economic levels and health technologies, for which most developing countries have increased per capita health expenditure [31, 32]. In summary, we can find that the above scholars mainly agree that environmental pollution leads to the decline of residents’ health and induces various physical and mental diseases, and in order to improve the quality of healthy life of residents, regional governments will increase the corresponding public health expenditures costs.

The above literature review provides a good theoretical basis for this paper to study the relationship among digital economic development, environmental pollution and residents’ health, but there is still room for further analysis: (i) Past studies have mainly focused on the relationship between two of them and few is about the relationship among the three. Besides, few scholars study the mediating role of environmental pollution between digital economy and residents’ health. (ii) In the study of the impact of digital economic development on the health of residents, digital economic development is more often measured by single indicators such as internet penetration, internet users and information technology use, which lacks comprehensiveness. Pitifully, as a new economic form, digital economy is still lack of unified standards to comprehensively measure its level of development. Zhao Tao et al. [33] argue that the measure indexes should include not only the digital infrastructure, but also other dimensions such as digital output, digital industry and digital application. (iii) Few scholars have studied the spatial effects of the digital economic development on residents’ health. Theoretically, the emergence of digital technology has weakened the limitations of spatial distance on the utilization of industrial resource endowment and residents’ health activities. What’s more, the application of digital technology provides real-time health consultation, information integration, medical consultation and other medical services for residents across regions, which realizes the spillover of knowledge and information, thus producing spatial spillover effects on the health of residents in the surrounding areas. Thus the results may be biased or incorrectly validated if the spatial spillover effect of digital economy development on residents’ health is not considered [34]. (iv) Due to differences in conditions such as geography, resource endowment, and traditional real economy, there is some variability in the level of digitization among regions in China. Zhang et al. [35] showed that regional economies positively influence regional digital technology infrastructure. To be specific, regions with high economic levels have higher efforts in building ICT infrastructure and introducing new digital technologies, and the infrastructure construction contributed to the overall local level of digital economy development [35]. However, it has seldom been investigated whether there are differences between regions in the impact of digital economy differences on residents’ health. Thus, an intermediary effect model and a spatial Durbin model are established for analysis in this paper. The mediation effect model can help us analyze the path between the digital economy and residents’ health, and the spatial Durbin model can help us better understand the impact of the digital economy and environmental pollution on the health of residents in neighboring areas.

**Fig. 1:**
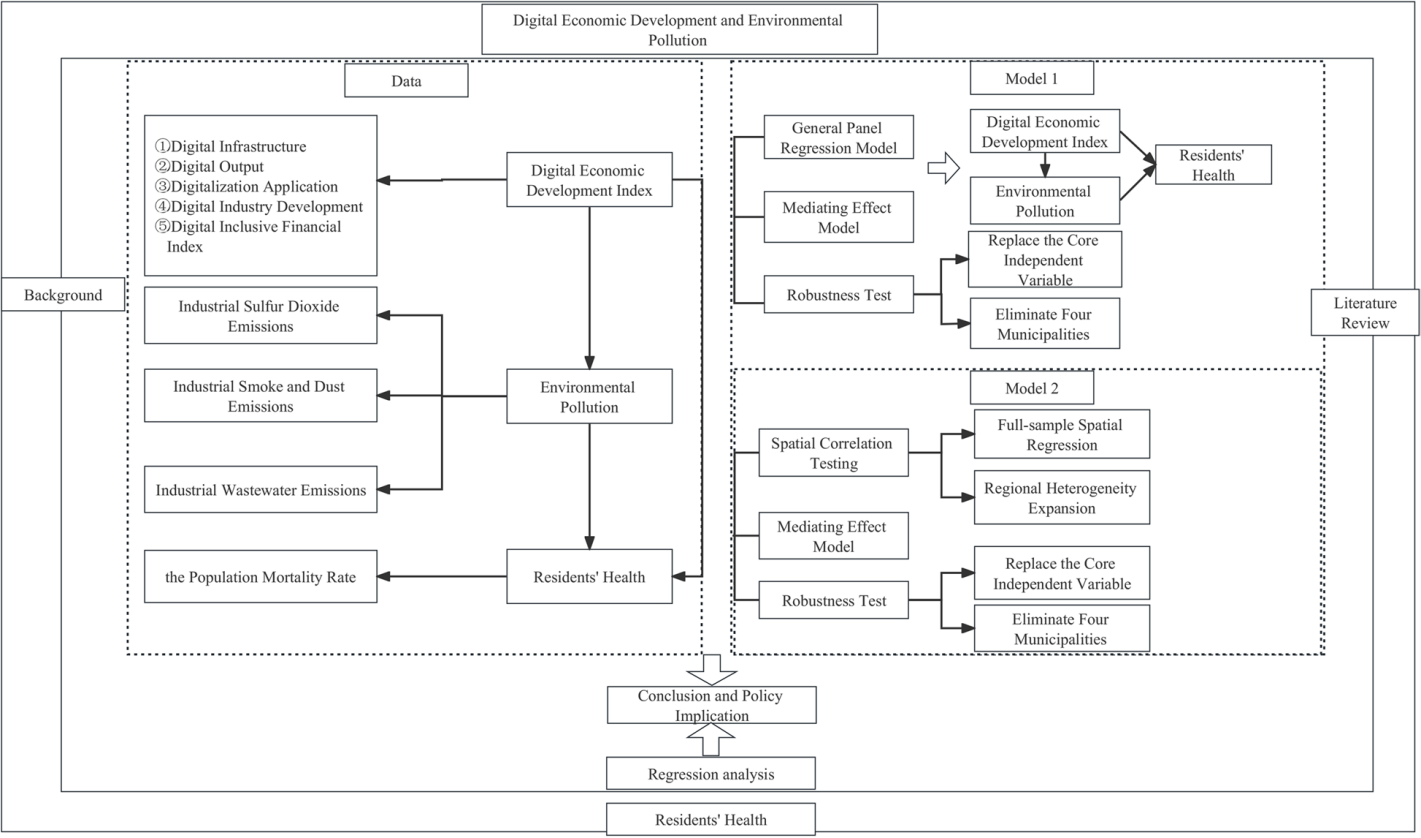
Research framework

The possible marginal contribution of this study goes as follows: (i) The comprehensive degree of digital economic development of prefecture-level cities is measured from five dimensions, including digital infrastructure, digital output, digital application, digital industrial development and digital inclusive finance, which enriches the scope of research related to the development of digital economy on the health level of residents. (ii) This paper ex-amines in detail the influence paths through which the development of the digital economy affects residents’ health, and determines the fact that residents’ health can be improved by means of solving the problem of environmental pollution, which is supplement to the existing literature. (iii) By means of Spatial Durbin model the paper analyzes the spatial effect of digital economic development on residents’ health in prefecture-level cities, and further explores the heterogeneity of digital economic development on residents’ health in eastern as well as central and western regions of China, in order to understand more comprehensively the impact of digital economic development on residents’ health. Figure 1 shows the research framework of our paper.

## Methods

### Study design

**Fig. 2:**
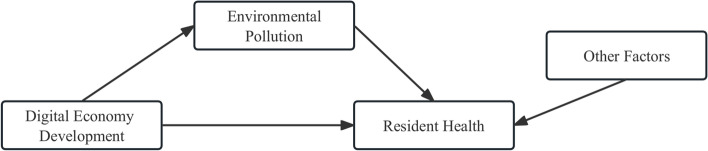
Relationship among digital economic development, environmental pollution and residents’ health

According to the above theoretical analysis, we propose the relationship between concepts as is shown in Fig. 2, which presents the main relationship among digital economic development, environmental pollution and residents’ health.

### Variable selection and data description

#### Dependent variable: health of the population (*HD*)

Due to the multidimensional nature of health, there is no unified standard to measure the health level of the nation’s residents, which is thus measured by many scholars according to such indicators as population mortality, life expectancy per capita, and neonatal mortality, etc. Referring to Maisonet M’s approach [36], we use the population mortality rate in this paper to represent the health level of residents in each region. The higher the population mortality rate, the worse the health condition of residents in that region.

#### Main independent variables: digital economic development (*DIG*)

The current academic measurement of *DIG* remains challenging because the digital economy is cross-overlapping with elements from multiple domains, which is difficult to measure and the classification criteria are not uniform [37]. At present, there are two ways to measure the level of the digital economic development. One is the index construction method, with which Xu constructs the index to measure the comprehensive level of urban digital economic development from the dimensions of digital economic index, digital industry development and digital infrastructure [38]; the other is to use the digital inclusive financial index designed by Guo et al. as an indicator to measure the level of local digital economy [39]. Compared with the empowerment method that relies on subjective judgment, on the basis of learning from Lulu Wang’s practice [40] and the availability of urban data, this paper attempts to use a more objective and comprehensive entropy value method to measure the development level of urban digital economy from five dimensions, including digital infrastructure, digital output, digital application, digital industry development and digital inclusive finance, etc. The meaning and weights of each indicator are shown in Table 1, in which the total index of digital inclusive finance includes three dimensions: the breadth of digital economy coverage, the depth of digital economy use and the degree of digitization. They are considered positive indicators.

**Table 1:** Digital economy index system and its weight

Secondary indexes	Three-level indexes	Weight
Digital infrastructure	Number of mobile phone users (per 10,000 people)	0.1892
Digital output	Telecom business volume (yuan)	0.1988
Digitalization application	Number of Internet broadband access users (per 10,000 households)	0.1943
Digital industry development	Number of employees in information service industry (person)	0.3603
Digital inclusive financial index	Peking University Digital Inclusive Finance Index	0.0574

First of all, in order to make the indicators of different dimensions comparable, this paper refers to the practice of Ma [41] and Wang [40] to standardize the 5 positive indicators:

1 $$X'_{\mathrm{ij}}=\frac{(X_j-X_{min})}{(X_{max}-X_{min})},1\leq i\;\leq\mathrm m,\;1\leq j\leq\mathrm n$$

Second, we need to determine the proportion of indicator *j* in city *i*:

2 $$P_{\mathrm{ij}}=\frac{{X'}_{ij}}{\sum_{i=1}^m{X'}_{ij}},\;0\leq{\mathrm P}_{\mathrm{ij}}\leq1$$

Further calculate the entropy value е_j_ of the value indicator *j* to obtain the information entropy redundant *d*_*j*_:

3 $$e_{j}=-\frac{1}{lnm}\sum\nolimits_{i=1}^{m}{p}_{ij}{lnp}_{ij}$$

4 $${d}_{j}=1-{e}_{j}$$

From (3) and (4), we can confirm that the weight of indicator *j* is *Wj*:

5 $${W}_{j}=\frac{{d}_{j}}{\sum\nolimits_{i=1}^{m}{d}_{i}}$$

Finally, the comprehensive level of the development of the digital economy in various cities is obtained:

6 $$DIG=\sum\nolimits_{i=1}^nW_jy_{ij},\;0\leq DIG\leq1$$

##### **Environmental pollution (*LNM*)**

For the variables of environmental pollution, some re-searchers measured the degree of environmental pollution from the multi-dimension of air, solid waste, and water resources [42], while other researchers measured it from a single perspective of air pollution [43]. With the existing studies as reference and in consideration of the comprehensiveness of environmental pollution at the urban level, this study selects a comprehensive index calculated with the three negative indicators of industrial sulfur dioxide emissions, industrial smoke and dust emissions and industrial wastewater emissions to characterize the degree of urban environmental pollution. The three indicators cover urban air pollution and water pollution, which can better address the endogenous problems of urban environmental pollutants on residents’ health. Since the summation-averaging method is easy to use, and the natural logarithm transformation can reduce or eliminate the heterosexuality of the data, this paper obtains the final urban environmental pollution measure by taking the logarithm on the basis of summing and averaging the industrial sulfur dioxide emissions, industrial soot emissions and industrial wastewater emissions.

### Control variable

(i) Education capital (*LNEDU*): The development of education in a region affects the level of digital economic development in the region, and other studies showed a positive relationship between individual education level and their health level [44]. Thus, in this study, the level of education capital is presented with the students of colleges and universities [45]. (ii) Population density (*LNDEN*): It is reasonable to select population density as a control variable in this study because in regions with large population, economic development will be better than that with small population, which makes it much easier to develop the construction of physical communication infrastructure which the development of digital economy is based on. Besides, studies have shown that cities with higher population density are more likely to have environmental pollution problems, and higher population density is associated with higher mortality rates [46]. (iii) The utilization of foreign capital (*LNFIS*): The actual utilization of foreign capital in the region (10,000 dollars as a unit) is selected for measurement because theoretically speaking, the more foreign investment introduced in a region, the more open the region is, which brings about the greater economic strength and the bigger investment in all kinds of medical services, hence the more health opportunities residents can enjoy and the better health conditions. (iv) Medical level (*HP*): Since higher medical level can provide effective protection for residents’ health [47], this paper selects the number of hospitals per ten thousand people in the region for measurement. In order to ensure the consistency of data, the above non-ratio indexes are processed by natural logarithm in this paper.

**Fig. 3:**
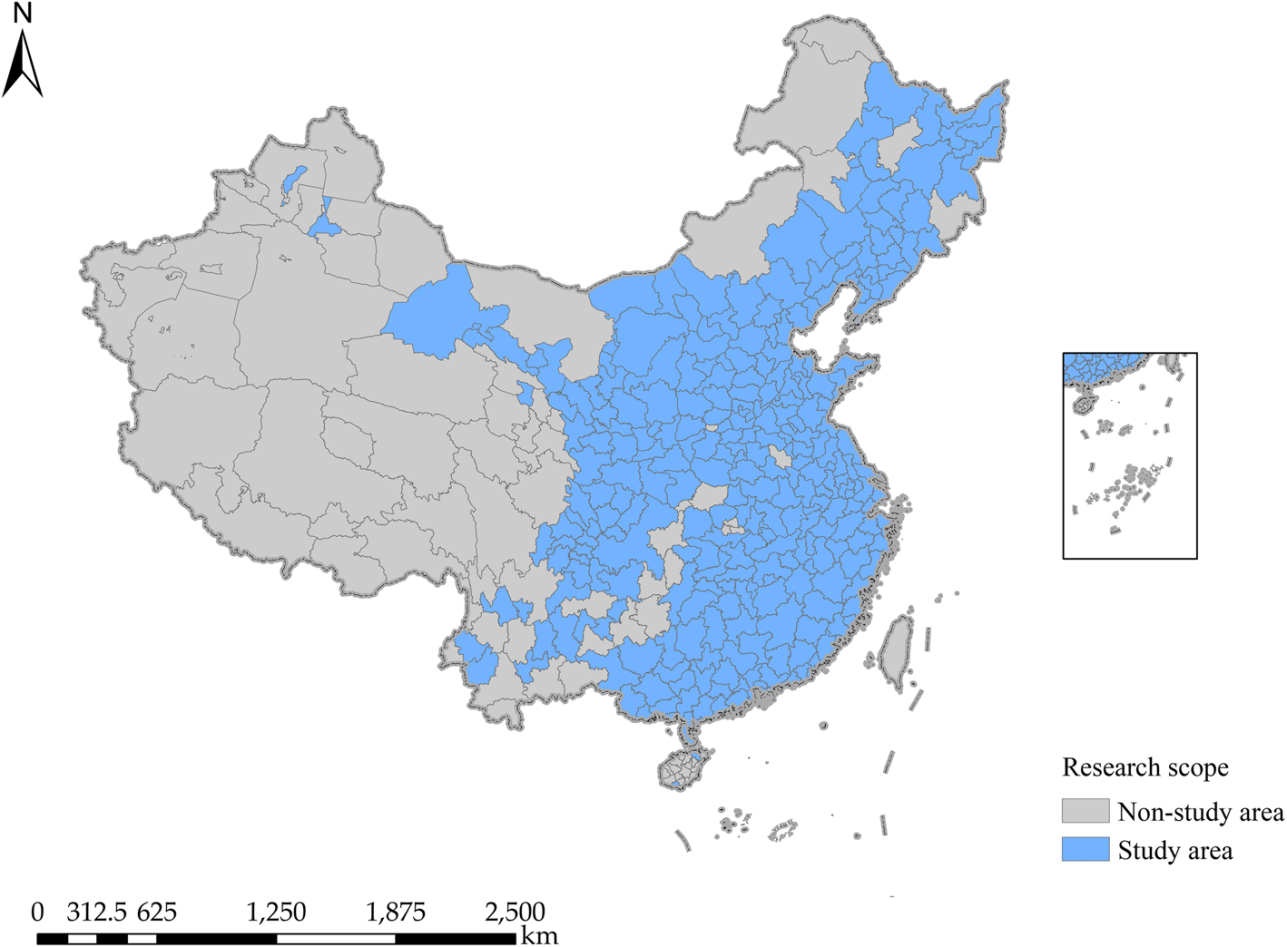
Study area map

Since China’s digital financial inclusion index was evaluated in 2011, and the core data of prefecture-level cities missing more after 2018, in the light of data availability and consistency, the data in this paper combines the adjustment of the latest administrative divisions in China in 2022 (a total of 293 prefecture-level cities are set), and panel data of 279 prefecture-level cities in China are selected as the sample for the empirical study during the period 2011–2017, so our total sample of 1 953 observation points; The map of the study area is shown in Fig. 3, in which the core data of prefecture-level cities such as Tibet, Qinghai, Hong Kong, Macao and Taiwan, and parts of Xinjiang are seriously missing values, and the study has not been carried out. The data are obtained from China Statistical Yearbook, China City Statistical Yearbook, China Environmental Statistical Yearbook and the regional annual National Economic and Social Development Statistical Bulletin by the National Bureau of Statistics (http://www.stats.gov.cn/); The data of the China Health Statistics Yearbook are obtained from the Health and Health Commission of the People’s Republic of China (http://www.nhc.gov.cn/); And the total digital inclusive finance index is obtained from the Digital Inclusive Finance Index released by the China Digital Finance Research Center of Peking University, which has been publicly available for free across the country through: pku_dfiic@163.com. The administrative map is obtained from the Resource and Environment Data Cloud Platform (http://www.resdc.cn). The descriptive statistics of variables are shown in Table 2.

**Table 2:** Descriptive statistics of variables (*N* = 1 953)

Abbreviations	Variable meaning	mean	Std. dev.	Min.	Max.
*HD*	Residents’ health	6.3604	2.0477	1.0300	22.0134
*DIG*	Digital economic development	0.0820	0.0691	0.0128	0.8355
*LNM*	Environmental pollution	1.0836	0.2977	0.6949	4.1978
*LNEDU*	Educational capital	10.3788	1.8297	0.000	13.8807
*LNDEN*	Population density	5.7724	0.9313	1.6292	10.9350
*LNFIS*	Utilization of foreign capital	10.6744	0.5995	8.7729	15.6752
*HP*	Medical level	0.5617	0.7852	0.0046	11.4505

### Model construction

#### Theoretical model

This paper employs the health production function theory created by Grossman as the basic theoretical model to analyze the health level of residents. The health production function takes into account economic, medical, educational and other factors [48], and this paper selects the level of digital economic development as the input factor of health production. In order to alleviate the endogenous problem of missing variables, the basic model is constructed by using double fixed effects as follows:

7 $$HD_{\mathrm{it}}=\alpha_0+\theta_1DIG_{\mathrm{it}}+\beta_1LNEDU_{\mathrm{it}}+\beta_2LNDEN_{\mathrm{it}}+\beta_3LNFIS_{\mathrm{it}}+\beta_4HP_{\mathrm{it}}+\mu_{\mathrm i}+\gamma_{\mathrm t}+\epsilon_{\mathrm{it}}$$

*HD*_it_ represents the health level of residents in prefecture-level city i in the year t. *DIG*_it_ represents the level of digital economic development of city i at time t. *LNEDU*_it_ represents the educational capital of city i in the year t. *LNDEN*_it_ denotes the population density of city i in year t. *LNFIS*_it_ represents the utilization of foreign capital in the year i. *HP*_it_ represents the medical level of city i in year t. α_i_ represents other influencing factors that cannot be exhausted. µ_i_ represents the regional fixed effect, which is used to control the characteristics that do not change with time. γ_t_ represents the year fixation effect, which is a unique feature used to control the time level that does not change with the individual. ε_it_ represents a random perturbation term.

### Mediating effect model

To examine the influence path between the development of digital economy and the health of the population, this study employs stepwise regression test to examine the mediating effect on the basis of Eq. (7). First, the estimated coefficient in the test formula (7) 𝜃_1_, if 𝜃_1_ is significant, indicates that the development of digital economy can affect the health of residents, indicating that the existence of intermediary effect is initially established. Second, the estimated coefficient 𝜃’_1_ of formula (8) and the estimated coefficient 𝜆_2_ for formula (9), if significant, indicate an indirect effect. Finally, the estimated coefficient of the test formula (9) is 𝜆_1_, if 𝜆_1_ is significant and 𝜆_2_ is significant, there is a direct effect, that is, there is a partial mediation effect; If 𝜆_1_ is not significant and 𝜆_2_ is significant, there is a complete mediation effect.

8 $$LNM_{\mathrm{it}}=\theta_0+\theta'_1DIG_{\mathrm{it}}+\theta_{\mathrm i}Z_{\mathrm{it}}+\mu_{\mathrm i}+\gamma_{\mathrm t}+\epsilon_{\mathrm{it}}$$

9 $$HD_{\mathrm{it}}=\beta_0+\lambda_1DIG_{\mathrm{it}}+\lambda_2LNM_{\mathrm{it}}+\lambda_{\mathrm i}Z_{\mathrm{it}}+\mu_{\mathrm i}+\gamma_{\mathrm t}+\epsilon_{\mathrm{it}}$$

Among them, *LNM*_it_ represents the intermediary variable of environmental pollution. *Zit* represents the control variable group at the city level. See Explanation for other variables (7).

### Spatial durbin model

In recent years, with the gradual improvement of spatial software tools such as ArcGIS and Geoda, spatial analysis has been increasingly used in public health and disease control. Using the spatial measurement methods and related tools can quickly grasp the spatial distribution and transmission path of diseases, accurately determine whether there are regional differences in disease occurrence, and quantitatively analyze the factors that cause differences. As the second largest economy in the world, China has made remarkable achievements in digital economy development and has also been able to better utilize digital technology for the benefit of population health, but unfortunately, there is still a spatial discrepancy between China’s digital economy [49] and the health of residents [50], which needs to be analyzed. Therefore, in light of the digital technology breaking the geographical distance limitation and the spatial spillover effect of digital economy development in prefecture-level cities on residents’ health, this paper introduces the spatial interaction terms of each variable on the basis of formula (7, 8 and 9), and the results of multiple tests show that spatial Durbin model (SDM) is more suitable than spatial error model and spatial lag model, thus the spatial Durbin model is constructed as follows:

10 $$HD_{\mathrm{it}}=\alpha_{\mathrm i}+\rho WHD_{\mathrm{it}}+\phi_1DIG_{\mathrm{it}}+\delta_1WDIG_{\mathrm{it}}+\beta_{\mathrm i}Z_{\mathrm{it}}+\delta_2WZ_{\mathrm{it}}+\mu_{\mathrm i}+\gamma_{\mathrm t}+\epsilon_{\mathrm{it}}$$

11 $$LNM_{\mathrm{it}}=\theta_{\mathrm i}+\rho'WLNM_{\mathrm{it}}+\eta_1DIG_{\mathrm{it}}+\delta'_1WDIG_{\mathrm{it}}+\eta_{\mathrm i}Z_{\mathrm{it}}+\delta'_2WZ_{\mathrm{it}}+\mu_{\mathrm i}+\gamma_{\mathrm t}+\epsilon_{\mathrm{it}}$$

12 $$HD_{\mathrm{it}}=\beta_{\mathrm i}+\rho"WHD_{\mathrm{it}}+\varphi_1DIG_{\mathrm{it}}+\lambda'_1WDIG_{\mathrm{it}}+\varphi_2LNM_{\mathrm{it}}+\lambda'_2WLNM_{\mathrm{it}}+\beta'_{\mathrm i}Z_{\mathrm{it}}+\delta'_3WZ_{\mathrm{it}}+\mu_{\mathrm i}+\gamma_{\mathrm t}+\epsilon_{\mathrm{it}}$$

In the above formula, $\rho$ is the elastic coefficient of the space lag term; W_ij_ represents spatial weight matrix, which mainly includes adjacency matrix, geographic matrix, economic matrix and economic geographic matrix. Since the adjacency matrix cannot help to determine the distance within the region and the imbalance of the digital economic development, and the geographic matrix does not deal with the relationship between regional economies, this study introduces the economic distance weight matrix as a spatial weight matrix to verify the spatial heterogeneity between the digital economy and residents’ health. Citing the basic principle of Bernard’s [51] document for reference, we combine the weight of geographical distance and economic distance to construct the weight matrix of economic distance. With the distance between city i and city j denoted as d_ij_, and PGDP_j_ and PGDP_i_ as the per capita GDP of city j and city i respectively, the element W_ij_ in the matrix is set as follows :

13 $$W_{ij}=\left\{\begin{array}{ll}0,&i=j\\\left(1/\left[{PGDP}_i-{PGDP}_j+1\right]\right)\times e^{-d\mathrm ij},&i\neq j\end{array}\right.\\$$

## Results

### Test results of mediating effect model

In this paper, the regional year double fixed effect model is selected, which can effectively control the regional heterogeneity and temporal trend effect. In addition, the statistical test level of this study was *α* = 0.05. The analysis results of the ordinary panel fixed effect model of the mediating effect of this study are shown in Models (7) ~ (9) of Table 3. Specifically, model (7) reports the regression results of the first stage benchmark model, which shows that the coefficient of the core independent variable the digital economic development is-8.7950, and it is significant at 1% level. Obviously, the development of digital economy has a negative inhibitory effect on the mortality rate of residents. That is, the development of digital economy promotes the improvement of residents’ health. Model (8) shows the regression relationship between the digital economy and environmental pollution in the second stage benchmark model. The result shows that digital economy has an indigenous negative effect on environmental pollution at 1% level, showing that for every unit of digital economy development, the degree of environmental pollution will decrease by 2.7632 units. With the intermediary variable *LNM* added in Model (9), the coefficient of the intermediary variable (*LNM*) is 0.3102, which is obvious at the level of 10%. The impact of digital economic development on population mortality was negatively correlated at 1% level with a coefficient of-9.6521, indicating that environmental pollution partially mediated the relationship between digital economic development and population health. In the regression results of the control variables, the population density (*LDEN*) and foreign capital utilization level (*LNFIS*) of models (7) and (9) significantly suppressed population mortality (both estimated coefficients were negative), indicating that both significantly promoted the resident health (*HD*) level. The results of ordinary panel regression analysis confirm that there are direct or indirect effects among the development of digital economy, environmental pollution and residents ‘health, and environmental pollution partly mediates the relationship between digital economy development and residents’ health.

**Table 3:** Test results of mediating effect model

Variable	Model (7)	Model (8)	Model (9)
	*HD*	*LNM*	*HD*
*DIG*	-8.7950^a^ (2.6462)	-2.7632^a^ (0.3090)	-9.6521^a^ (2.8329)
*LNM*			0.3102^c^ (0.1693)
*LNEDU*	0.0121 (0.0530)	0.0004 (0.0031)	0.0123 (0.0529)
*LNDEN*	-0.2649^b^ (0.0987)	0.0202^a^(0.0053)	-0.2587^b^ (0.0996)
*LNFIS*	0.6612^b^ (0.2224)	-0.0581^b^(0.0274)	-0.6792^b^ (0.2297)
*HP*	0.0618 (0.0674)	0.0174^a^ (0.0042)	0.0672 (0.0672)
_Cons	20.0001^a^ (2.3861)	3.4600^a^ (0.2411)	21.0739^a^ (2.6844)
Fixed Effect	Double fixed effect	Double fixed effect	Double fixed effect
*N*	1 953	1 953	1 953
*R*^*2*^	0.3430	0.7219	0.3440

^a^, ^b^, and ^c^, respectively, indicates significance at the 1, 5, and 10% level. Robustness standard error in Parentheses

### Robustness test

Due to the influence of the selection of measurement indicators on the research conclusion, this paper conducts the robustness test through the following two methods to ensure the reliability of the research conclusion. The first method is to replace the core independent variable of this paper with the Urban Inclusive Financial Index (*LNDIG*_*2*_) released by the Digital Finance Research Center of Peking University. Urban Inclusive Finance Index includes the breadth of digital coverage, the depth of digital use and the degree of digitization, among which the depth of digital use includes payment, insurance, monetary fund, credit services, investment and credit, which can reflect the degree of digital technology utilization and the depth of digital economy development in a city. The estimated results are shown in Table 4. The coefficient symbols and significance of the core independent variables and the intermediary variables were basically consistent with the regression results in Table 3, and both were significant at the 5% level, proving that the study results were relatively robust and reliable. The second method is to eliminate four municipalities directly under the central government and provincial capitals in order to reduce the data error caused by the large urban grade gap. The estimated results are shown in Table 5. The coefficient symbols and significance of core independent variables and intermediary variables are basically consistent with the regression results in Table 3, and all are significant at the 5% level, proving that the results are relatively robust.

**Table 4:** Robustness test of replacing core independent variables

Variable	(7) *HD*	(8) *LNM*	(9) *HD*
*LNDIG*_*2*_	-0.2753^c^ (0.1418)	-0.1259^a^ (0.0292)	-0.2416^b^ (0.0932)
*LNM*			0.4930^b^ (0.1848)
_Cons	17.8968^a^ (2.6769)	2.6828^a^ (0.3184)	10.639^a^ (1.2882)
Control Variable	Control	Control	Control
*Fixed Effect*	Double fixed effect	Double fixed effect	Double fixed effect
*N*	1 953	1 953	1 953
*R*^*2*^	0.3385	0.7107	0.3386

^a^, ^b^, and ^c^, respectively, indicates significance at the 1, 5, and 10% level. Robustness standard error in Parentheses

**Table 5:** Excluding robustness test of four municipalities

Variable	(7) *HD*	(8) *LNM*	(9) *HD*
*DIG*	-14.5693^a^ (3.0179)	-3.3461^a^ (0.3429)	-16.0673^a^ (3.2858)
*LNM*			0.4477^b^ (0.1778)
_Cons	14.4049^a^ (2.1559)	2.2512^a^ (0.2411)	15.4127^a^ (2.2812)
Control Variable	Control	Control	Control
*Fixed Effect*	Double fixed effect	Double fixed effect	Double fixed effect
*N*	1 925	1 925	1 925
*R*^*2*^	0.3469	0.7092	0.3481

^a^, ^b^, and ^c^, respectively, indicates significance at the 1, 5, and 10% level. Robustness standard error in Parentheses

### Spatial correlation testing

In theory, the test of Moran’s I (Moran’s index) and scatter plot can be conducted by means of *stata.16* software to determine whether a factor has spatial characteristics. Table 6 is the Moran index of digital economic development and residents’ health, which shows that when the global Moran’s I index is tested for the digital economic development of 279 prefecture-level cities, the Moran’s I indexes from 2011 to 2017 are greater than 0 and pass the significance test at the level of 1%, and it is the same case with the global Moran’s I index test of the residents’ health level in prefecture-level cities. The above results show that there is a positive spatial correlation between digital economic development and residents’ health in prefecture-level cities, which is suitable for spatial econometric analysis (see Table 6).

**Table 6:** Moran ' s I results

Time	*DIG*		*HD*	
	Moran’s I	*p*	Moran’s I	*p*
2011	0.1230	0.0000	0.1790	0.0000
2012	0.1200	0.0000	0.0950	0.0000
2013	0.1030	0.0000	0.1900	0.0000
2014	0.1120	0.0000	0.1900	0.0000
2015	0.1000	0.0000	0.1060	0.0000
2016	0.0870	0.0000	0.1580	0.0000
2017	0.0955	0.0000	0.1212	0.0000

**Fig. 4:**
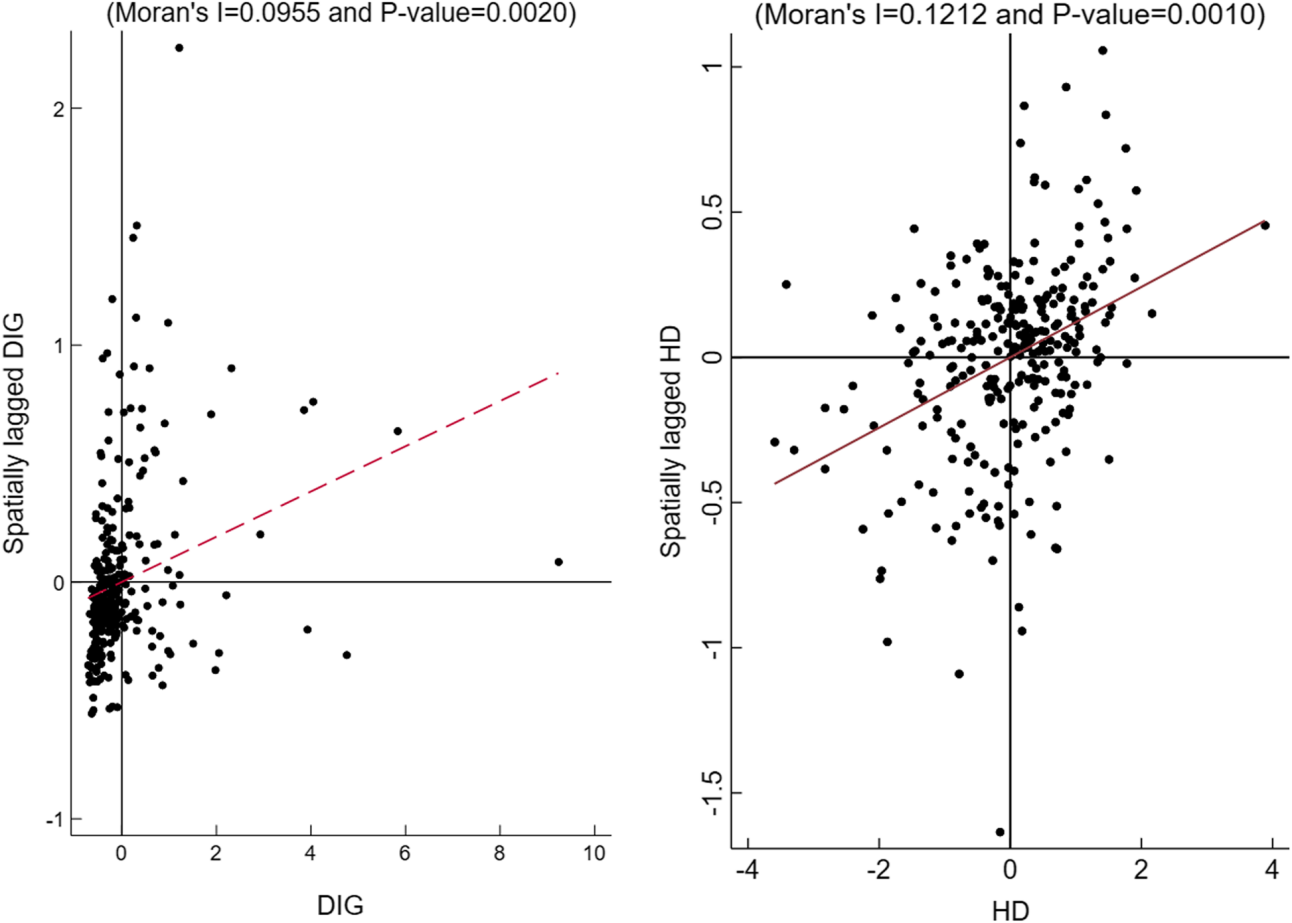
Moran scatter plot of China ' s digital economic development and residents ' health in 2017

Because the global correlation test cannot be used to describe the local atypical features, this study selects the Moran scatter plot of digital economic development and residents’ health in 2017 in order to further analyze the spatial agglomeration characteristics of the digital economic development and residents’ health in prefecture-level cities in China. Among the four quadrants of Moran scatter diagram, the first one represents high-high value, the second one represents low-high value, the third one represents low-low value, and the fourth one represents high-low value. According to the Moran scatter plot in Fig. 4, the vast majority of prefecture-level cities in Fig. 4 are in the third quadrant of the digital economic development level, namely, low-low value agglomeration, which indicates that the digital economic development level of the city itself is low, so are the digital economic development levels of the surrounding cities. Other cities are in the second quadrant which shows that the city is at a low level of digital economic development but the surrounding cities at a high level and the fourth quadrant which shows the opposite. The scatter diagram of residents’ health shows that the residents’ health in 279 prefecture-level cities mainly presents a low-low value, indicating that the residents’ health level of the city as well as the surrounding cities is low; In addition, the residents’ health level in some prefecture-level city is scattered in the second and fourth quadrants (Fig. 4). And some cities are in the second quadrant, indicating that the health level of residents in these cities is low but that of residents in surrounding cities is high. Thus, the local Moran index scatter plot further shows that China’s digital economic development and residents’ health level indicate spatial heterogeneity as well as spatial agglomeration which is not absolute and complete.

**Fig. 5:**
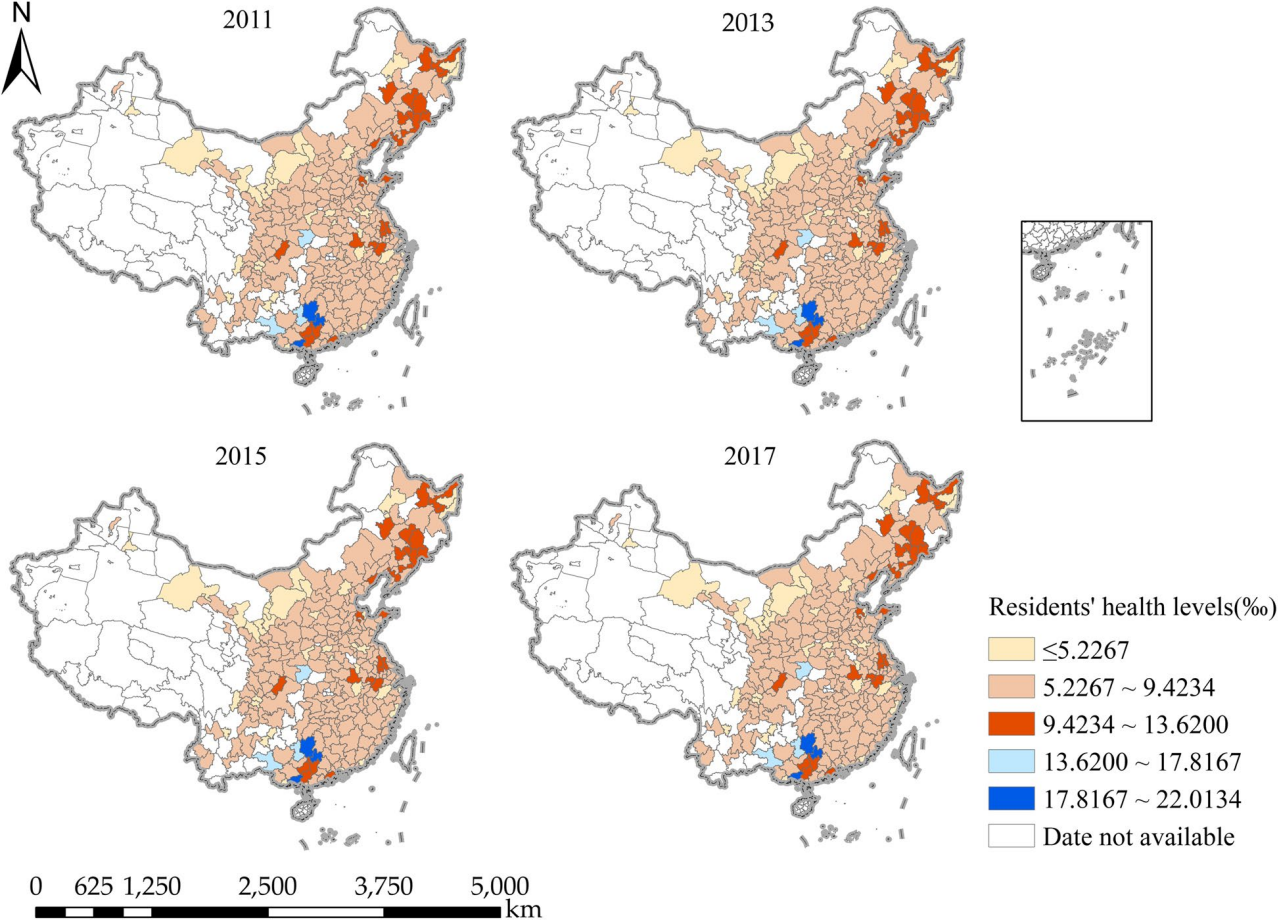
Spatial distribution maps of healthy prefecture-level cities in China in 2011, 2013, 2015 and 2017

In order to more intuitively display the residents’ health distribution in the study area, the residents’ health data of cities in China in 2011, 2013, 2015 and 2017 were selected as sample data, and visualized by mapping using ArcGIS 10.2 software. In the paper, we divided the population mortality data into five grades using an equal division method: high (17.8167 ~ 22.0134), slightly higher (13.6200 ~ 17.8167), medium (9.4234 ~ 13.6200), slightly lower (5.2267 ~ 9.4234), and low (≤ 5.2267). From Fig. 5, we found that in this study, the health level of residents in 279 prefecture-level cities in China did not have obvious temporal sequence change but showed relative spatial agglomeration phenomenon. The health level of residents in most prefecture-level cities is at a high level and concentrated distribution, especially in the central and eastern regions. Surprisingly, the health level of residents in some prefecture-level cities is at a slightly lower level, such as the northeast and south China. Thus, this phenomenon once again verifies that the spatial agglomeration characteristics of the health level of Chinese residents are not absolute and complete, and there is still a certain regional heterogeneity (see Fig. 5).

### Spatial durbin regression results

Since the previous part of this paper has verified the existence of the spatial effect of digital economy development and residents’ health, it is necessary to establish a spatial panel regression model to analyze the relationship between China’s digital economy and environmental pollution and residents’ health, which can better explain the spatial distribution characteristics of residents’ health and the degree of influence of related factors. Before spatial econometric analysis, LM test is needed to test whether the data is suitable for spatial regression model or ordinary panel regression model (OLS). The three test LM (the statistic is 347.5270, *P* < 0.05) and R_LM (the statistic is 288.8300 ,*P* < 0.05) values of the spatial error model reject the original assumption. The *P* value of LM test for spatial lag model rejects the original assumption (the statistic is 62.9240, *P* < 0.05), and the R_LM test of spatial lag model also reject the original hypothesis (the statistic is 4.2270, *P* < 0.05). These results just show that there are spatial error items and spatial lag items in the data, so spatial Durbin model analysis should be conducted (see Table 7). On the basis of it, Wald test and likelihood ratio LR test are further conducted to verify whether the SDM model will degenerate into SLM or SEM model. The results in Table 7 show that the statistics of Wald-error and Wald-lag are 13.8200 and 12.0600 respectively, and that of the LR-error and LR-lag are 13.7100 and 12.0500 respectively, which indicates the passing of the 5% dominance test. Therefore, the null hypothesis that SDM degenerates into SLM or SEM is rejected and this study employs the SDM model for spatial econometric analysis. In addition, it is necessary to test the fixed effect and random effect of the model, with the model selected by Hausman test, the result of which in this study shows that the coefficient is 55.4500, which rejects the null hypothesis of random effects at a significance level of 5%. Then, at the significance level of 10%, the time fixed effect and the individual fixed effect were tested separately, and the results showed that the coefficient of the time fixed effect test was 657.1000, and the *P* value was less than 0.05 (rejecting the null hypothesis); the coefficient of the regional fixed effect test was 23.0300 and the *P*-value was 0.0106 (rejecting the null hypothesis), so it was more appropriate to select a double fixed-effects model for regression analysis. And the specific results were shown in Table 7.

**Table 7:** LM, LR, Wald and Hausman test results

Test	Statistical quantities	*P*-value
LM-error	347.5270	0.0000
Robust LM-error	288.8300	0.0000
LM-lag	62.9240	0.0000
Robust LM-lag	4.2270	0.0000
Wald spatial error	13.8200	0.0168
Wald spatial lag	12.0600	0.0339
LR spatial error	13.7100	0.0176
LR spatial lag	12.0500	0.0341
Hausman	55.4500	0.0000

To reduce the effect of heteroscedasticity, we apply the robustness standard error to the final results of the spatial Durbin model. According to Table 8, after introducing the spatial weight matrix of economic geographic distance, the development of digital economy can directly act on the health of residents and environmental pollution, but environmental pollution only partially mediates the relationship between the development of digital economy and the health of residents in neighboring areas. Firstly, model (10) represents the impact of the digital economy on the health of residents, from which we find that whether it is a local effect or a spatial spillover effect, the development of the digital economy has a significant inhibitory effect on the local and adjacent population mortality (*DIG* coefficient is -4.4279, and the *P* value is meaningful at the statistical level of 10%; W*DIG* coefficient is -20.0938, *P* < 0.05). Secondly, the model (11) represents the impact of the digital economy on environmental pollution, from which we find that the digital economy has direct effect and spatial spillover effect on urban environmental pollution (*DIG* coefficient: -0.6727, *P* < 0.05; W*DIG* coefficient: -1.3986, *P* value is significant at the statistical level of 10%). Finally, Model (12) represents the impact of the digital economy on the health of residents after the inclusion of environmental pollution as a mediating variable. As for the local effect, the digital economy has a significant impact on the health level of local residents, and there is no significant impact on environmental pollution (*DIG* coefficient: -0.6271, *P* < 0.05; *LNM* coefficient: 0.1228, *P* > 0.05). As for the spatial spillover part, the spatial lag coefficient of digital economic development in SDM model is significantly negative, and the coefficient is -19.1321, which is significant at the level of 5%, which shows that with spatial lag into con-sideration, the mortality rate of residents in adjacent prefecture-level cities decreases by 19.1321 units accompanied with each unit of digital economic development. Besides, environmental pollution partially mediates the impact of the digital economy on the health of residents in neighboring areas (W*LNM* coefficient is 0.3129, and the *P* value is significant at the statistical level of 10%). Summarizing the results of the spatial model, we find that the digital economy has a significant contribution to the health of residents in both local and neighboring areas, but after the inclusion of mediation variables (*LNM*), environmental pollution does not have a mediating effect in the impact of the digital economy on the health of local residents; In contrast, environmental pollution is a mediating variable in the digital economy affecting the health of residents in neighboring areas.

**Table 8:** Empirical results

Variable	SDM Model (10)	SDM Model (11)	SDM Model (12)
	*HD*	*LNM*	*HD*
*DIG*	-4.4729^c^ (2.6894)	-0.6727^b^ (0.2534)	-0.6271^b^ (0.2410)
*LNM*			0.1228 (1.0248)
W*DIG*	-20.0938^b^ (8.4114)	-1.3986^c^ (0.7911)	-19.1321^b^ (8.4268)
W*LNM*			0.3129* (0.1852)
*ρ*	0.6681^a^ (0.0464)	0.3881^a^ (0.0627)	0.6679^a^ (0.0465)
Control Variable	Control	Control	Control
Fixed Effect	Double fixed effect	Double fixed effect	Double fixed effect
*N*	1 953	1 953	1 953
*R*^*2*^	0.0503	0.2390	0.0479

^a^, ^b^, and ^c^, respectively, indicates significance at the 1, 5, and 10% level. Robustness standard error in Parentheses

Since the spatial interaction coefficient cannot directly reflect the marginal impact of explanatory variables, this paper decomposes the spatial spillover effect of digital economic development in order to accurately evaluate the effect of digital economic development on residents’ health level and spatial spillover effect (see Table 9). The direct effects of digital economy and environmental pollution on the health of local residents were not significant, but had significant indirect effects on the health of residents in adjacent areas. Specifically, after the addition of the intermediary variable (*LNM*), the digital economy and environmental pollution in the direct effect do not have an impact on the health of local residents at a statistical level of 5% (*DIG* coefficient: -5.0195, *LNM* coefficient: 0.0508). On the contrary, the indirect effect of digital economy and environmental pollution has a significant impact on the health of residents (*DIG* coefficient is -64.2219, *P* < 0.05; *LNM* coefficient: 1.0535, *P* value is meaningful at the statistical level of 10%), indicating that compared with the direct effect, the indirect effect of digital economy development contributes more, and under the indirect effect of environmental pollution, the digital economy still has an impact on the health of residents in neighboring prefecture-level cities. It shows that the digital economy can improve the health of residents in neighboring areas by alleviating environmental pollution.

**Table 9:** Direct effect, indirect effect and total effect analysis and mediating effect regression results (*N* = 1 953)

Variable	SDM Model (10) *HD*			SDM Model (11) *LNM*			SDM Model (12) *HD*		
	Direct Effect	Indirect Effect	Gross Effect	Direct Effect	Indirect Effect	Gross Effect	Direct Effect	Indirect Effect	Gross Effect
*DIG*	-5.7600^b^ (2.8808)	-70.2502^b^ (26.3576)	-76.0102^b^ (27.4713)	-0.7014^b^ (0.2622)	-2.6223^b^ (1.3060)	-3.3236^b^ (1.3726)	-5.0195 (2.6848)	-64.2219^b^ (26.1525)	-69.2414^b^ (27.0514)
*LNM*							0.0508 (0.0396)	1.0535^c^ (0.5727)	1.1042^c^ (0.5885)
*ρ*	(0.0464) 0.6681^a^	(0.0464) 0.6681^a^	(0.0464) 0.6681^a^	(0.0627) 0.3881^a^	(0.0627) 0.3881^a^	(0.0627) 0.3881^a^	(0.0465) 0.6679^a^	(0.0465) 0.6679^a^	(0.0465) 0.6679^a^
Control Variable	Control	Control	Control	Control	Control	Control	Control	Control	Control
Fixed Effect	Double fixed effect	Double fixed effect	Double fixed effect	Double fixed effect	Double fixed effect	Double fixed effect	Double fixed effect	Double fixed effect	Double fixed effect
*R*^*2*^	0.0503	0.0503	0.0503	0.2390	0.2390	0.2390	0.0479	0.0479	0.0479

^a^, ^b^, and ^c^, respectively, indicates significance at the 1, 5, and 10% level. Robustness standard error in Parentheses

In addition, in order to verify the reliability of the SDM model results, this paper also conducts the robustness test by replacing explanatory variables and excluding the four major municipalities. As shown in Table 10, column 1 to 3 represents the robustness test of replacing explanatory variables with the Inclusive Financial Index (*LNDIG*_*2*_), and column 4 to 6 represents the robustness test of excluding the four municipalities(*DIG’*). The results show that the data of each variable changes slightly, but the sign and significance of the core independent variable and the spatial term coefficient (ρ) were basically consistent with the results of the SDM model (10), (11) and (12) in Table 8, respectively,which proves that the results of the SDM model are robust and reliable.

**Table 10:** Spatial Durbin robustness test

Variable	SDM Model (10) *HD*	SDM Model (11) *LNM*	SDM Model (12) *HD*	SDM Model (10) *HD*	SDM Model (11) *LNM*	SDM Model (12) *HD*
*LNDIG*_*2*_	-0.3371^b^ (0.1400)	-0.0662^c^ (0.0390)	-1.6778^b^ (0.4973)			
*DIG’*				-4.3316^b^ (1.3238)	-0.0791^b^ (0.0391)	-4.7279^a^ (1.3505)
*LNM*			0.4180 (0.3745)			0.5673 (0.3755)
W *LNDIG*_*2*_	-1.5990^a^ (0.4972)	-0.1977^c^ (0.1066)	-0.3484^b^ (0.1398)			
W*DIG’*				-37.9363^a^ (10.8245)	-0.0708^c^ (0.4087)	-41.2776^a^ (11.0119)
W*LNM*			0.2859^b^ (0.1048)			0.2548^b^ (0.1067)
*ρ*	0.6533^a^ (0.0472)	0.3534^a^ (0.0581)	0.6413^a^ (0.0481)	0.6470^a^ (0.0533)	0.3654^a^ (0.0654)	0.6351^a^ (0.0540)
Control Variable	Control	Control	Control	Control	Control	Control
Fixed Effect	Double fixed effect	Double fixed effect	Double fixed effect	Double fixed effect	Double fixed effect	Double fixed effect
*N*	1 953	1 953	1 953	1 925	1 925	1 925
*R*^*2*^	0.0446	0.2501	0.0409	0.0566	0.3144	0.0544

^a^, ^b^, and ^c^, respectively, indicates significance at the 1, 5, and 10% level. Robustness standard error in Parentheses

### Analysis of regional heterogeneity expansion

Due to the differences in geographical environment, resource endowments and economic development policies, there is an inevitable imbalance of digital economic development and residents’ health level among prefecture-level cities. According to the criteria of eastern, central and western regions divided by the National Bureau of Statistics of China, and on the basis of Yin [52] and Zhu [53], this paper empirically examines the impact of the development of China in the eastern regions and central and western regions (see Table 11). After the sample region, the empirical results are basically consistent with those of the whole region of China. However, while the spatial spillover effect of digital economy on residents’ health in the central and western regions is more obvious than that in the eastern region. Specifically, the results after the inclusion of the mediation variables showed (at a statistical level of 5%) that there was no significant effect on the health of local residents in both the digital economy and environmental pollution in the eastern region (*DIG* coefficient: -6.4789; *LNM* coefficient: 0.3566) in the local effect. The digital economy in the Midwest region has a significant inhibitory effect on the mortality rate of the resident population (*DIG* coefficient: -0.5584, *P* < 0.05), but environmental pollution is not the mediating variable between them (*LNM* coefficient: 1.7412, *P* > 0.05). In contrast, in the spatial spillover effect, digital economy and environmental pollution in both the eastern and central western regions have a significant effect on the health of residents in neighboring prefecture-level cities. It is worth noting that the spatial spillover effect of digital economy and environmental pollution in the central and western regions is greater than that in the eastern region, where the *DIG* coefficient in the central and western regions is -42.3074 and the *LNM* coefficient is 0.3506; the *DIG* coefficient in the eastern region is -7.8818, and the coefficient of *LNM* is 0.2050.The above results show that after considering the mediating effect of environmental pollution, compared with the eastern region, the digital economy in the central and western region has a more significant role in promoting the health of adjacent urban residents.

**Table 11:** Regional heterogeneity regression results

Variable	The eastern region			The central and western regions		
	SDM Model (10) *HD*	SDM Model (11) *LNM*	SDM Model (12) *HD*	SDM Model (10) *HD*	SDM Model (11) *LNM*	SDM Model (12) *HD*
*DIG*	-8.3704 (8.2601)	-0.7951^a^ (0.2261)	-6.4789 (8.5785)	-0.7386^c^ (0.3868)	-0.0633^b^ (0.0223)	-0.5584^b^ (0.2678)
*LNM*			0.3566 (0.6437)			1.7412 (4.4573)
W*DIG*	-8.4300^b^ (3.1202)	-2.1853^a^ (0.6017)	-7.8818^b^ (3.1591)	-43.1761^b^ (13.7320)	-0.0420^c^ (0.0243)	-42.3074^b^ (13.7155)
W*LNM*			0.2050^b^ (0.0948)			0.3506^b^ (0.1508)
*ρ*	0.2569^a^ (0.0833)	0.3018^a^ (0.0792)	0.2250^a^ (0.0852)	0.7001^a^ (0.0425)	0.2647^a^ (0.0730)	0.7006^a^ (0.0428)
Control Variable	Control	Control	Control	Control	Control	Control
Fixed Effect	Double fixed effect	Double fixed effect	Double fixed effect	Double fixed effect	Double fixed effect	Double fixed effect
*N*	700	700	700	1253	1253	1253
*R*^*2*^	0.0273	0.2262	0.0202	0.0799	0.2304	0.0881

^a^, ^b^, and ^c^, respectively, indicates significance at the 1, 5, and 10% level. Robustness standard error in Parentheses

## Discussion

By changing economic and social life, the digital revolution creates for a country and a region economic opportunity across spatial and temporal boundaries [54], an opportunity that may bring adverse effects on the urban environment due to the consumption of power energy, or may reduce environmental pollution through innovative means such as new technologies, and can provide a new service model for each health care facility. Thus, this study attempts to study the panel data of 279 prefecture-level cities in China from 2011 to 2017, and uses the mediating effect model to explore the relationship and influence path between digital economic development, environmental pollution and residents’ health, and further analyzes the spatial effects and regional heterogeneity of digital economy development on residents’ health by employing the spatial Durbin model. The research findings are as follows.

The ordinary panel regression results can be viewed from three aspects. Firstly, the development of digital economy can promote the health of the population, which is not difficult to understand that the expansion of Industry 4.0 technology in the field of health has brought a good economic foundation and development opportunities to personalized medicine, precision medicine and telemedicine [55]. Global lockdowns during the COVID-19 pandemic have highlighted the adverse effects of social isolation and isolation on the physical and mental health of residents, and digital technologies can break down this information silos and provide targeted health services to residents to reduce the risk of disease [56]. More importantly, the use of digital technologies can help strengthen the governance capacity of health sectors in low- and middle-income countries, improve health efficiency, and ultimately narrow the digital health divide between developed and developing countries, as Haron et al. [57] have shown that the use of digital technologies has facilitated early screening for oral cancer in South and South-East Asia. Thus, the digital economy has a promoting effect on the health level of residents. Secondly, the development of digital economy can significantly reduce urban environmental pollution. Our environment is flooded with air pollutants such as SO_2_ and NO_2_, which are generally considered to be the main sources of pollution in the industrial sector [58], and traditional means such as regulating environmental pollution at the policy level cannot effectively alleviate the emission of industrial pollution; However, the digital economy, an important feature of Industry 4.0, has become the core driving force of China’s new and old kinetic energy, and its promotion promotes the digital development of China’s industries and brings possibilities for China’s environmentally sustainable development [59]. To be specific, enterprises and other pollutant emission subjects can, by means of digital technology such as VR (virtual reality) and IOT (internet of things), effectively integrate various types of information resources in production decision making and alleviate the problems of information fragmentation and asymmetry, which can help to efficiently advance the production process of enterprises, thus improving their production efficiency and supporting ecofriendly development by reducing resource waste [60, 61]. This kind of technical support also helps to reduce pollution notably for such emerging markets as Brazil, China, and India [8]. What’s more, the transformation of technology-enabled environmental governance model has become the consensus of environmental supervision departments across China. Digital China Construction and Development Process Report (2019) points out that the data management technology of ecological environment has become an important means to promote the modernization of environmental governance system and governance capacity, and that the national environmental monitoring network continues has been sound all the time, providing accurate support for joint prevention and control as well as supervision and law enforcement of pollution [62]. Finally, the current study has found that environmental pollution partially mediated the relationship between digital economic development and residents’ health, which indicates that the development of digital economy and environmental pollution can independently or cooperatively affect residents’ health level. The conclusion of this study further verifies the close relationship among the three.

According to the spatial regression results of the full sample in China, we found that the digital economy has a significant role in promoting the health of local and neighboring residents, but after the inclusion of environmental pollution mediation variables, environmental pollution is not a mediating variable between the digital economy and the health of local residents; In contrast, environmental pollution is a mediating variable in the digital economy affecting the health of residents in neighboring areas. The current study suggests that is related to the degree of digitization of the region and the digital literacy of the residents. To be specific, residents in regions with a higher degree of digitization have higher digital literacy [35], and are more willing to get access to health information and healthcare services by means of internet platforms and information technology tools than those in regions with a lower degree of digitization [63]. The development of the digital economy has led to the development of local healthcare, and the higher the level of local digital healthcare, the more capable it is of providing more accurate internet healthcare services, which makes it easier to attract residents from other areas to come to the hospital, creating a “siphoning effect” of local hospitals on internet healthcare resources from neighboring areas, which to some extent leads to the phenomenon of local residents’ health resources being squeezed while the positive health effects of residents from neighboring areas are more significant. However, from an overall perspective, digital infrastructure such as the internet, as a carrier to accommodate but not limited to health knowledge, can achieve the goal of improving residents’ personal health literacy and health self-management level through the effective use of health information contained in the internet. That is, the development of the digital economy with internet as the carrier is conducive to promoting the overall improvement of residents’ health. At present, Industry 4.0 is expected to realize the material flow of circular economy through data, accelerate the “symbiosis of digital economy and real economy”, promote the upgrading of industrial structure, promote green total factor production efficiency, and improve environmental quality [64, 65]. The effective integration of the digital economy and the real economy can not only achieve environmental benefits, but also promote the improvement of the health level of residents, so it is necessary to establish a platform for the interconnection of all walks of life to support the entire innovation process and solve problems related to society as a whole [66]. However, most studies have focused more on the relationship between the digital economy and environmental pollution or the health of residents, and our results show that the development of the digital economy can mitigating environmental pollution to improve the health of residents in neighboring prefecture-level cities, so previous research focusing on the relationship between the two is not enough.

According to the results of regional spatial heterogeneity expansion in China, after the inclusion of environmental pollution intermediary variables, the digital economy in the eastern China as well as central and western China has a promoting effect on the health of residents in neighboring prefecture-level cities, and it is worth mentioning that compared with the spatial spillover effect in eastern China, the development of digital economy and environmental pollution have a more obvious spatial spillover effect on the health of residents in the central and western regions. According to the “pollution shelter” hypothesis, some polluting industries in developed countries with higher environmental standards have moved to other developing countries in line with global cooperation, resulting in these developing countries becoming “pollution havens“ [67]. It should be noted that in China, there are signs of pollutant transfer from the eastern region to the central and western regions, and this change has made the negative effects of environmental pollution in the central and western regions on the health of residents more prominent [68]. Integrating digital technology into traditional industries and creating China Industry 4.0 will effectively reduce the health hazards caused by pollution transfer. At the same time, the concept of Industry 4.0 will be extended to the field of health and the realization of China Health 4.0, which will also help to break the geographical restrictions and time constraints of traditional economic and medical services, and alleviate the imbalance between regions in which residents enjoy medical services. Specifically, in the aspect of digital technology promoting the development of medical and health services, residents in economically underdeveloped areas (such as the Midwest China) have late-development advantage. On the one hand, less economically developed regions have late-development advantage of digital innovation in the health industry. Daviedo’s law points out that in the era of digital economy, enterprises with innovation ability and new products are more likely to occupy the dominant position in the market for a long time. That is, there is Matthew effect [69]. Then, with the innovation and application of digital technology, polarization of digital economy may occur in the region. Developed regions have a higher level of digital economic development, and existing health services industries have taken advantage of digital technology to occupy market space. In order to maintain the source of health resources, leading enterprises may vigorously suppress emerging health services enterprises. Medical institutions that take the lead in providing accurate services by employing digital technology may be more attractive to patients, which will make it difficult for emerging health enterprises and other medical institutions to carry out health care services to a certain extent. On the contrary, economically underdeveloped regions have broad market space and more innovation and entrepreneurship activities, so they can refer to the existing experience and combine the characteristics of local health industry to provide innovative health care services. In this way, the health promotion effect of digital technology on residents in economically underdeveloped regions may be more obvious. On the other hand, residents in economically underdeveloped regions have the late-developing advantage of learning health knowledge. Due to the spillover and sharing of digital technology, economically underdeveloped areas can learn from the successful experience. By vigorously promoting the application and integration of digital technology in the medical and health service industry, information chasing and health digitization can be realized, and the health promotion of local residents can be accelerated.

### Shortcomings and future research

Different from previous studies that only focus on the relationship among internet use or information technology, environmental pollution and residents’ health, this study measures the level of the digital economy at the urban level in China from five dimensions: digital infrastructure, digital output, digital application, digital industry development and digital inclusive finance, and explores the relationship among digital economic development, environmental pollution and residents’ health through panel data, which enriches to a certain extent the research approach of the digital economic development affecting residents’ health. However, the current study still falls short in the following three aspects: (i) This paper comprehensively measures the impact mechanism of digital economy on residents’ health from five dimensions, but fails to conduct a more detailed analysis in each single dimension; Due to the limitations of our research themes, we have not conducted a more detailed study of the role path between the digital economy and environmental pollution, for example, we have mentioned in the introduction and literature that the digital economy may improve environmental quality through some intermediary paths (such as industrial structure upgrading, innovation efficiency, etc.), and follow-up research can be carried out in this regard. (ii) This paper emphasizes the direct effect, indirect effect and regional heterogeneity of digital economic development in space, but fails to study the heterogeneity of digital economic development on residents’ health in different countries or different social demo-graphic characteristics. Hopefully, the follow-up research can be carried out in the two respects. (iii) Our study sample did not cover all regions of China, and according to Fig. 3, the reader can find that some cities in central and western China did not consider it due to a lack of data. In empirical testing, it also affects the effectiveness of regional heterogeneity testing. In the future, we can continue to fully mine this data for in-depth research.

## Conclusion

Based on the panel data of 279 prefecture-level cities in China from 2011 to 2017, this study empirically tests the causal relationship and influence path between digital economic development, environmental pollution and residents’ health in prefecture-level cities in China by means of intermediary effect model and spatial Durbin model, and reveals the heterogeneity of the impact of digital economic development on residents’ health in different regions. The result shows: firstly, the development of digital economy can promote the health of urban residents not only directly, but also indirectly by reducing environmental pollution; secondly, there is a positive relationship between the development of urban digital economy and the spatial spillover effect of residents’ health in China, but under the mediation of environmental pollution, the digital economy only has a significant positive impact on the health of residents in neighboring areas; thirdly, after the inclusion of environmental pollution variables, the development of the digital economy in central and western China has a more significant role in promoting the health of residents in neighboring cities compared with the eastern region. Therefore, this urged the prefecture level government continue to vigorously promote the digital economy become a new engine of economic development, for example, on the macro to continue to implement the strategy of “Internet +”, using satellite Internet and industrial digital resources, speed up the innovation industry digital and the integration of new forms of energy industry, build China’s industrial 4.0 pattern to reduce environmental pollution; On the micro level, the government should set up an information support fund to support the application of various digital technologies in the medical field and the manufacturing industry from the perspective of capital investment to improve the system and information service capacity. And should continue to optimize the upper design, provide different technical guidance, for example, the government needs to pay attention to the digital divide between regions, through the developed regions and less developed areas of knowledge technology exchange platform, narrow the gap between regional digital economy development and digital medical services, create a good environment for development for residents' health.

## Data Availability

The original data can be found on the official websites of the National Bureau of Statistics of China (http://www.stats.gov.cn/), the Health and Health Commission of the People’s Republic of China (http://www.nhc.gov.cn/), the public total digital inclusive finance index can be freely available from the China Digital Finance Research Center of Peking University (pku_dfiic@163.com), the Resource and Environment Data Cloud Platform (http://www.resdc.cn ).
